# Inkjet Printing of an Electron Injection Layer: New Role of Cesium Carbonate Interlayer in Polymer OLEDs

**DOI:** 10.3390/polym13010080

**Published:** 2020-12-28

**Authors:** Amruth C, Beata Luszczynska, Wassima Rekab, Marek Zdzislaw Szymanski, Jacek Ulanski

**Affiliations:** Department of Molecular Physics, Faculty of Chemistry, Lodz University of Technology, 90-924 Lodz, Poland; amruth.c@ucalgary.ca (A.C.); wassima.rekab@p.lodz.pl (W.R.); marek@marekszymanski.com (M.Z.S.)

**Keywords:** printed PLEDs, inkjet printing, cesium carbonate, interlayer, emission area patterning, printed/organic/flexible electronics

## Abstract

Among solution-processable techniques, inkjet printing is a potential method for manufacturing low-cost and high-resolution polymer organic light-emitting diodes (PLEDs) for displays/solid-state lighting applications. Herein, we demonstrate use of the inkjet printed cesium carbonate (Cs_2_CO_3_) film as an electron injection interlayer. We have elaborated the Cs_2_CO_3_ ink using an alcohol-based solvent for the industrial-grade printhead. The printed Cs_2_CO_3_ layer morphology was investigated by means of an optical microscope and an atomic force microscope. The PLEDs based on emissive polymer (Super Yellow) with printed Cs_2_CO_3_ interlayer show a remarkable current efficiency and luminance compared to the PLEDs made without the Cs_2_CO_3_ layer. Such results suggest that the Cs_2_CO_3_ is a promising material for the formulation of the electron injecting inkjet inks. The possibility of inkjet printing of an efficient electron injecting layer enables in situ patterning of PLEDs’ emission area. Such a simple and flexible technique can be applied for a wide range of applications such as signage, pictograms, advertising, smart packaging, etc.

## 1. Introduction

Organic light-emitting diodes (OLEDs) are already being used to manufacture displays and lighting because of their features, such as being lightweight, mechanically flexible, and having high color contrast [1,2,3]. They are currently produced via a vacuum process, which is an expensive technique and involves high material wastage. Therefore, the displays, particularly the large panels, are still costly [4]; thus, reducing manufacturing costs is crucial in this field. Solution-processing techniques, specifically, printing methods, offer low-cost and straightforward procedure to produce OLEDs. Furthermore, the use of organic polymers as an active layer assists in the formulation of stable ink and achieving high-quality printed film when compared to inks and films formed with the small molecule organic material s [5]. Notably, the inkjet printing is of great interest for being a non-contact, drop-on-demand, and roll to roll production system [6]. For this reason it is employed in the fabrication on a laboratory scale of many organic electronic devices, namely photovoltaics [7], organic thin-film transistors [8], polymer organic light-emitting diodes (PLEDs) [9] and sensors [10]. Nevertheless, many challenges need to be addressed before employing the printing technology to produce electronics on a large scale [11]. For instances, the problems include: ink formulation, creating homogeneous thin films, and layer by layer printing [12,13,14].

A simple PLED structure consists of an emissive layer (EML) sandwiched between two electrodes, namely, anode and cathode. Such simple PLEDs are easy to fabricate, but they result in poor performances and short lifetimes. To enhance the PLEDs performances usually inter-layers are employed between the electrodes and EML. The interlayers placed between cathode and EML improve electron injection and/or transport properties, and when they are placed between an anode and EML improve hole injection and/or transport properties [15]. However, fabrication of a multi-layer PLEDs through the solution process, specifically inkjet printing, is challenging since the subsequent layer deposited via the printing process can damage the previously deposited layer. Therefore, layers should be printed using orthogonal solvents onto the underlying layer [16] or the underlying layer should be crosslinked if similar solvents are used [17].

The lack of printable electron injection layer (EIL) materials [18,19,20,21] limits the realization of fully printable PLEDs. In most of the published works a low work function metals such as calcium and barium covered by aluminum have been used as cathodes [22,23,24]. However, these low work function metals are unstable, and using them in the formulation of ink is impossible. Alternatively, a thin layer of LiF or CsF has also been used as an EIL [25,26,27,28]. Nonetheless, these materials are insoluble in most of the solvents used in the inkjet process, and thus they are not suitable for the printing technique.

On the other hand, the thin film of inorganic salt: cesium carbonate (Cs_2_CO_3_) serves as an efficient EIL in PLEDs [29,30,31]. The exciting feature of the Cs_2_CO_3_ is that it can be deposited using both thermal evaporation and spin-coating process [32,33,34]. Huang et al. have reported that the OLEDs fabricated with spin-coated and thermal evaporated Cs_2_CO_3_ layers showed similar performance [35]. These authors have also shown that solution-processed Cs_2_CO_3_ chemically reacts with the deposited Al cathode and forms a low work-function interfacial layer. The Cs_2_CO_3_ is able to oxidize the first few layers of the deposited Al cathode and forms an Al–O–Cs structure which reduces the work function of the cathode. Moreover, in both cases of thermal evaporation and solution processing, the effect of the low work-function interfacial layer formation has the same source: the formation of an Al–O–Cs structure at the interface. Moreover, Hasegawa et al. found that the cesium carbonate injects electrons in combination with metals like Al, Ag, and Au [36]. This allows the fully printed cathode system made of printed cesium carbonate as EIL and subsequently printed Ag as cathode to be realized [37,38,39]. Moreover, Cs_2_CO_3_ is much cheaper (~16 USD for 5 g in Sigma.com, date December 2020) than other commercially available solution processable electron injection materials and therefore, suitable for producing low-cost devices. Such materials are also interesting for solar cell applications [40,41]. Although Cs_2_CO_3_ layers have been successfully demonstrated for organic electronics devices, no sufficient attention has been paid to the ink formulation and depositing it via inkjet printing technique.

The PLEDs’ high-end applications include display screens for television and mobile phones [42], whereas low end applications include smart packaging [43], smart greeting cards [44], and signage [45,46]. PLEDs pixels for display screen should be printed with high precision and need to be studied to achieve high-quality pixels through the inkjet. On the other hand, the low-end applications do not need displays made from high-density pixels; however, defining only the PLEDs’ emission area (EA) with desired shapes and dimensions would be adequate. Usually, the EA in PLEDs is defined by patterning the electrodes; however, this conventional patterning process is expensive, and it involves complex processes such as photolithography and etching. Alternatively, Donggeon et al. have demonstrated patterning of EA of OLEDs via screen printing and spray coating of the dielectric layer [47]. Nevertheless, such a patterning method still requires specially designed masks. Thus, it adds more processing steps, which increase the complexity and the cost of the PLEDs’ production. Moreover, the EA resolution is limited by the mask in screen printing and spray-coating processes.

In this work, we present the inkjet printing of Cs_2_CO_3_ thin film for PLEDs applications. We discuss the Cs_2_CO_3_ ink formulation process and optimize the inkjet parameters to obtain stable drops. The stable jetting was received after tuning the waveform parameters, which drives the printhead. Before the printing of Cs_2_CO_3_ films, the wetting behavior of the polymeric emissive layer studied through the contact angle measurements are discussed. The printed film’s morphologies were examined by an optical microscope and atomic force microscope (AFM). The electron injection capability of the printed Cs_2_CO_3_ films was examined by incorporating it in the PLEDs with a polymer emissive layer. Furthermore, we have demonstrated a method in which electrons’ injection could be precisely controlled by selectively deposited Cs_2_CO_3_. Such an approach allows easy patterning of the emission area of the PLEDs, which could be implemented in various printed electronic applications. According to our best knowledge, this is the first report where inkjet printing of the Cs_2_CO_3_ layer is studied, and used in PLED as EIL and patterning of the PLEDs’ emission area.

## 2. Materials and Methods

### 2.1. Materials

Toluene (anhydrous, 99.8%), 2-ethoxyethanol (ReagentPlus, 99%), cesium carbonate and aluminum (Al) were purchased from Sigma Aldrich, St Louis, MO, USA. Super Yellow (SY, poly (para-phenylene vinylene) copolymer) was purchased from Merck Ltd., Germany. Poly(3,4-ethylenedioxythiophene):poly(styrenesulfonate) (PEDOT:PSS) was received from Heraeus, Germany (CLEVIOS P VP AI 4083). Indium-tin-oxide (ITO) coated glass substrates, which have a sheet resistance of 20 Ω/square, were purchased from Ossila Ltd., Sheffield, UK.

### 2.2. Instruments

The inkjet printer, PIXDRO LP50, was supplied by Meyer Burger Technology Ltd., Thun, Switzerland. In our study, the spectra S-class printhead (Model—SE-128 AA) was used, which operates on a piezoelectric principle [48]. The viscosity of the ink was measured using a HAAKE Viscotester 7 plus supplied by Thermo Fisher Scientific, Karlsruhe, Germany. During the measurement the rotation speed and the temperature were kept at 200 rpm and 25 °C, respectively. The surface tension and the contact angle were measured using the OCA 15EC Goniometer provided by DataPhysics Instruments GmbH, Filderstadt, Germany. Bruker Dektak 2D profilometer received from Bruker Ltd., Coventry, UK was used to measure the thickness of the constituent layers of PLEDs. A Flex-Axiom nanosurf AFM purchased from Nanosurf GmbH, Langen, Germany was used to study the surface morphology of the printed layers. A Keithley 2400 source measure unit was used to measure the current density vs. voltage characteristics of PLEDs. A Minolta spectroradiometer supplied by Konica Minolta Sensing Americas, Inc., NJ, USA was used to measure the luminance of PLEDs. A MicroHR spectrometer and a CCD (Charge-Coupled Devices) camera 3500 (Horiba JobinYvon) was used to measure the electroluminescence (EL) spectra.

### 2.3. Polymer Organic Light-Emitting Diode (PLED) Fabrication

The PLED structure consists of: glass/ITO (100 nm)/PEDOT:PSS (30 nm)/Super Yellow (SY, 70 nm)/ EIL (Cs_2_CO_3_)/Al (100 nm). The fabrication of PLEDs was a multi-step process. At the first step, the ITO-coated substrates were cleaned with acetone and isopropyl alcohol in an ultrasonic bath, followed by treatment with oxygen plasma for 90 s. The substrates were then spin-coated with PEDOT:PSS, as a hole injection layer (HIL), at the spin speed of 2000 rpm for 50 s. The coated films were immediately annealed at a temperature of 200 °C for 10 min on a hotplate to remove residual solvent. Then, the super yellow polymer solution (5 mg/mL in toluene) was spin-coated as an emissive layer (EML) at the speed of 1500 rpm for 60 s, followed by annealing at a temperature of 90 °C for 30 min. Subsequently, the Cs_2_CO_3_ solution was inkjet printed, followed by annealing at 120 °C for 30 min. All the above steps were carried out in ambient conditions. Finally, Al as a cathode was deposited using a vacuum thermal evaporator, with a deposition rate of 0.5 nm/s. The area of the devices was 3 mm × 1.5 mm. To compare the performance of printed Cs_2_CO_3_ PLEDs with the conventional devices, we have fabricated four different PLEDs just by varying the cathode system: (a) only evaporated Al, (b) evaporated Ca/Al, (c) evaporated Cs_2_CO_3_ (2 nm, indicated by quartz thickness monitor)/Al and (d) spin-coated Cs_2_CO_3_ /Al.

## 3. Results

### 3.1. Ink Formulation and Jetting Characteristics

In inkjet printing, the rheological parameters of inkjet ink: the density, the viscosity and the surface tension are highly crucial in the drop formation process [49]. These parameters can be arranged in one dimensionless figure of merit i.e., the inverse of Ohnesorge number (Z) expressed as:

(1) $$Z=\frac{a\gamma \rho^{1/2}}{\eta }$$

where “*a*” is the radius of the nozzle, “*γ*” is the ink surface tension, “ρ” is the ink density and “*η*” is the ink viscosity [50]. Many reports show tremendous efforts to estimate the range of Z number over which the inks are printable. Reis et al. reported that the Z number of ink ranges from 1 to 10 results in the printable ink [51]. Recently, Jang et al. suggested that the Z number range from 4 to 14 [14] is characteristic for the printable ink. Therefore, from the literature findings, one can assume that the inks having Z numbers between 1 and 14 are appropriate for the stable drop formation process.

**Figure 1:**
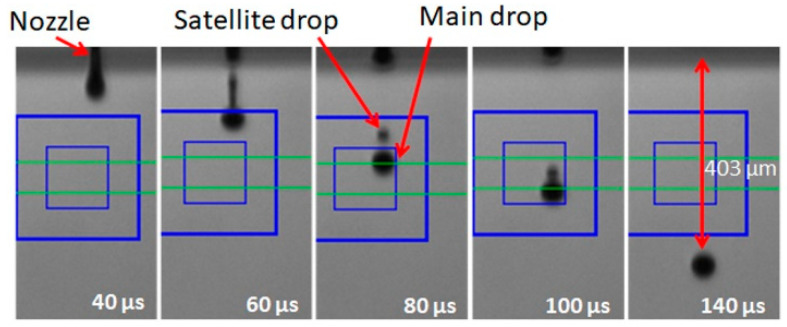
Pictures of drop formation of Cs_2_CO_3_ ink. The volume and the velocity of drop at 140 µs are, respectively, 21 pL and 2.88 m/s.

In our study, we have formulated the ink by dissolving of the cesium carbonate in 2-ethoxyethanol. The physical parameters of Cs_2_CO_3_ ink (2 mg/mL) are presented in Table 1. One can notice that the value of the Z number in our case was 22.4, which is far beyond the recommended range between 1 and 14. Nevertheless, in spite of such high Z value, we received the spherical drops after tuning the waveform, which drives the piezo-head. Figure 1 shows the sequential images of the drop formation process of Cs_2_CO_3_ ink. It was observed that the stable spherical drop was formed at a time of 140 µs. It is worth mentioning that the stable drop formation for the inks having a Z number higher than 14 has been already reported elsewhere [52,53]. For example, Torrisi et al. reported drop formation for the graphene ink with Z number equal to 24 [53]. Shin et al. achieved the printable ethylene glycol–water mixture ink with Z number at the level of 35.5 by applying a double waveform to control the drop formation [52]. Therefore, we claim that the range 1 < Z < 14 is not a strictly required condition for a stable drop formation process in an inkjet.

### 3.2. Ink Wettability

**Figure 2:**
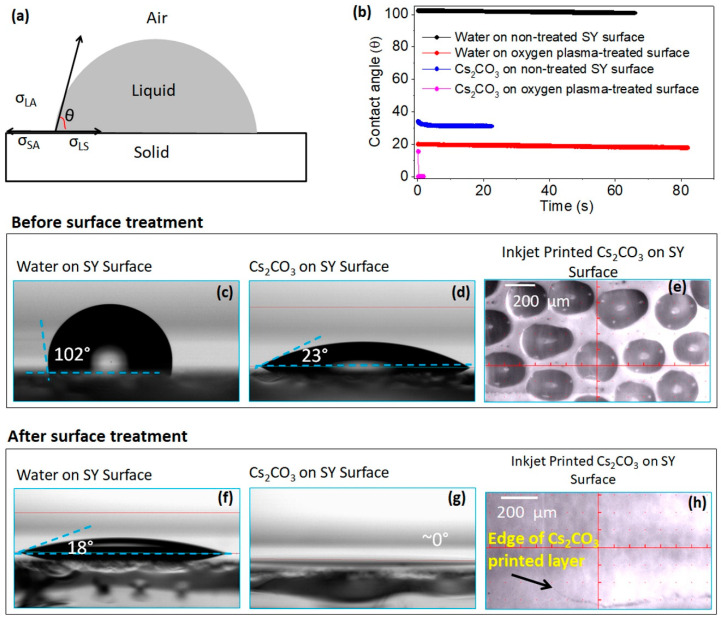
(**a**) A schematic illustration of a drop on solid surface. $\sigma_{\mathrm{SL}}$ $\sigma_{\mathrm{SA}}$, and $\sigma_{\mathrm{LA}}$ are three interFigure 2. CO_3_ solution on both non-treated and oxygen plasma-treated surface. (**b**) Contact angle before surface treatment of (**c**) Water (**d**) Cs_2_CO_3_ ink on the SY (Super Yellow, poly (para-phenylene vinylene) surface. (**e**) Inkjet printed Cs_2_CO_3_ layer on SY layer before surface treatment. Contact angle after surface treatment of (**f**) water (**g**) Cs_2_CO_3_ ink on SY surface. (**h**) Inkjet printed Cs_2_CO_3_ layer on SY after surface treatment.

The wettability of a drop on a solid surface plays a significant role in the film formation process [54,55]. The degree of wettability of a drop can be estimated by measuring the contact angle, which is the result of three interfacial tensions: solid–liquid (σ_SL_), solid–air (σ_SA_) and liquid–air (σ_LA_) [56]. Schematic in Figure 2a shows the contact angle (θ), which is the angle between solid/liquid interface and tangent to the liquid/air interface.

Initially, we measured the contact angle of the water (the universal solvent) to examine the nature of the SY surface. The water drop on the SY surface shows a contact angle of 102° (Figure 2c), which indicates that the surface is hydrophobic. The contact angle of Cs_2_CO_3_ ink on the SY surface shows a contact angle of 23°, as shown in Figure 2d. When this ink was printed, the SY surface inhibits the drop spreading, resulting in formation of droplet islands, seen in Figure 2e, leaving the patches/marks of Cs_2_CO_3_ (Figure S1). Although Cs_2_CO_3_ ink has shown some wettability of the SY surface, it is still not enough to evenly cover the entire surface. Therefore, it was necessary to modify the SY surface to improve the ink wettability.

Oxygen plasma is commonly used to treat the surface, especially to decrease the hydrophobicity of the surface [57]. The water contact angle θ measured on the oxygen plasma treated SY surface fell to 18°, as shown in Figure 2f. For the cesium carbonate ink the θ angle was found to be nearly 0° (Figure 2b,g), which indicates complete wetting. Figure 2h shows the microscopic image of printed cesium carbonate on the oxygen plasma-treated SY surface that reveals a tendency to spread the drops uniformly. For this reason, here we have used oxygen plasma treatment to modify the SY surface before printing Cs_2_CO_3_ ink.

### 3.3. Optimization of Cs_2_CO_3_ Ink Concentration to Attain Best PLEDs in Terms of Performance

The inks with various concentrations of Cs_2_CO_3_: 0.5, 1, 2, and 3 mg/mL dissolved in 2-ethoxyethanol were examined to produce high-quality thin layers. We prepared a series of PLEDs with the structure: glass/ITO/PEDOT:PSS/SY/Cs_2_CO_3_/Al, where the Cs_2_CO_3_ layer was printed at various print resolutions: 100 dpi to 800 dpi in an interval of 100 dpi. The unit of print resolution is dots per inch (dpi).

As expected, basing on literature [30,31,35], we have found that the printed Cs_2_CO_3_ interlayer assists in injecting the electrons. The PLEDs with Cs_2_CO_3_ layer always performed much better than the PLEDs without Cs_2_CO_3_, as is shown in the Table 2. Plots of current density–voltage, luminance–voltage, and current efficiency–luminance characteristics are presented in Supplementary Materials (Figure S2). The best PLEDs in terms of luminance and current efficiency were obtained when the print resolution was either 500 dpi or 600 dpi. The turn-on voltage of all PLEDs was found to be between 4 V and 5 V. It was found that the PLEDs printed with the Cs_2_CO_3_ ink with 2 mg/mL concentration show the best performance; therefore, this ink was used for further study.

### 3.4. Influence of Print Resolution on Cs_2_CO_3_ Film Formation and on PLEDs’ Performance

**Figure 3:**
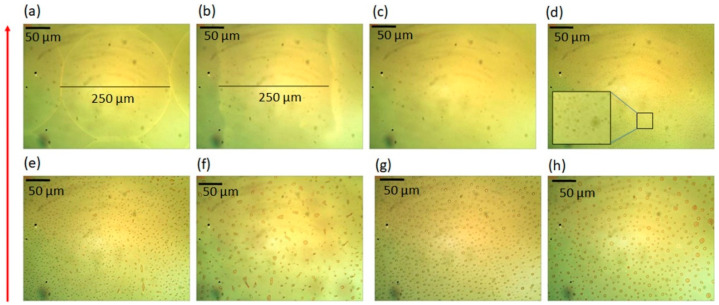
Optical images of Cs_2_CO_3_ films printed using ink with 2 mg/mL concentration with resolutions: (**a**) 100 dpi (**b**) 200 dpi (**c**) 300 dpi (**d**) 400 dpi (**e**) 500 dpi (**f**) 600 dpi (**g**) 700 dpi and (**h**) 800 dpi. Red arrow on the left side shows the printing direction (vertically up).

**Figure 4:**
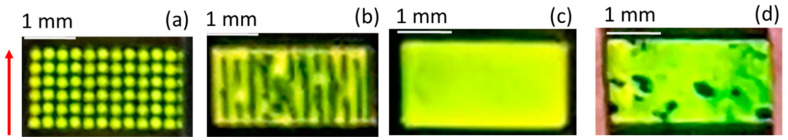
Light emission from PLEDs, where Cs_2_CO_3_ film was printed with various resolutions (**a**) 100 dpi (**b**) 200 dpi (**c**) 300 dpi to 600 dpi (**d**) 700 dpi and 800 dpi. Red arrow on the left side shows the printing direction (vertically up).

The print resolution significantly influences the film formation process as it is directly associated with the amount of ink being dispensed; the density of deposited ink drops increases with increasing print resolution. For example, films printed with 2 mg/mL concentration at resolution of 100 dpi contains 420 μg/inch^2^ of Cs_2_CO_3_ and at 200 dpi the films contain 820 μg/inch^2^ of Cs_2_CO_3_ and so on. Figure 3 shows optical images of Cs_2_CO_3_ films printed at various resolutions: 100 dpi to 800 dpi with an interval of 100 dpi. When ink is printed at the resolution of 100 dpi, the film is composed of circular Cs_2_CO_3_ droplets because the drop diameter is equal or less than the distance between the neighboring drops, as shown in Figure 3a. The PLEDs with such Cs_2_CO_3_ interlayers emit light only from the regions where the Cs_2_CO_3_ was deposited, as shown in Figure 4a, whereas areas without the Cs_2_CO_3_ layer did not emit the light.

When the print resolution increased to 200 dpi, the Cs_2_CO_3_ drops merge along the printing direction. But they remain partially separated in the perpendicular direction, and one can see a line formation in printing direction, as shown in Figure 3b. As a result, the PLEDs fabricated with the corresponding films showed discontinuous emission, as shown in Figure 4b.

For print resolutions 300 dpi and higher, the printed droplets merge in all directions forming continuous films. However, the optical images (Figure 3c–h) reveal that these films are composed of micron-sized particles, and these particles grow in size with an increased print resolution, i.e., with an increasing amount of deposited ink. One can assume that this is the result of Cs_2_CO_3_ crystallization due to prolonged drying time (at least 30 to 60 s after printing). The Ostwald ripening process could explain the formation of such relatively large Cs_2_CO_3_ particles/crystals wherein the unstable small particles are “swallowed” by bigger particles [58]. Looking at lighting PLEDs with the naked eye, the PLEDs with the light-emitting layer produced with the resolutions of 300 dpi, 400 dpi, 500 dpi, and 600 dpi exhibit uniform emission, as shown in Figure 4c. However, the PLEDs with printed active layers at higher resolutions (700 dpi and 800 dpi) show the emission area with dark spots, as seen in Figure 4d. The presence of such dark spots may be attributed to the large Cs_2_CO_3_ crystals that are too thick to provide an effective injection of the charge carriers.

**Figure 5:**
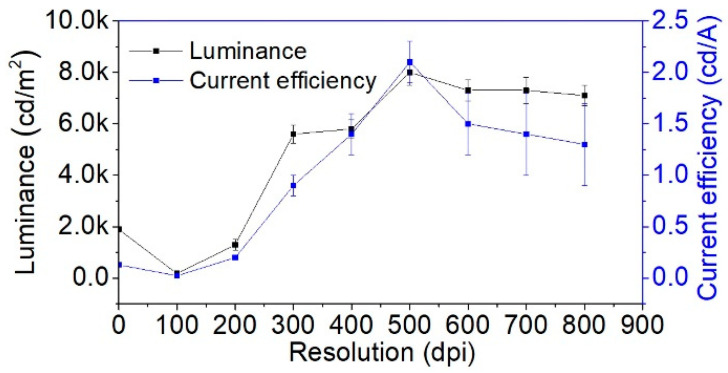
Luminance and current efficiency of PLEDs with the Cs_2_CO_3_ interlayer printed with different resolutions: 100 dpi, 200 dpi, 300 dpi, 400 dpi, 500 dpi, 600 dpi, 700 dpi and 800 dpi.

The performances of PLEDs fabricated with a printed Cs_2_CO_3_ layer (using the ink with 2 mg/mL concentration) at various print resolutions are shown in Figure 5 (the PLEDs characteristics can be found in Figure S3). The luminance and the current efficiency ascend with the increase in the resolution and reach maxima at 500 dpi. Then, they gradually decline, indicating that 500 dpi is the optimal print resolution. This trend of PLEDs performance might be attributed to the nature of film morphology formed at a different resolution. For instance, at low resolutions of 100 dpi and 200 dpi, the drops were not well connected to form a uniform film; thus, the luminance and current efficiency are far lower than the peak value. For the resolution between 300 dpi and 600 dpi, the performance might mostly depend on the crystals’ size and distribution. At a higher print resolution, 700 dpi, and 800 dpi, the non-uniform light emission (Figure 4d) might be responsible for the reduced performance.

### 3.5. Atomic Force Microscopy (AFM) Study of Cs_2_CO_3_ Film Printed at Various Print Resolutions

**Figure 6:**
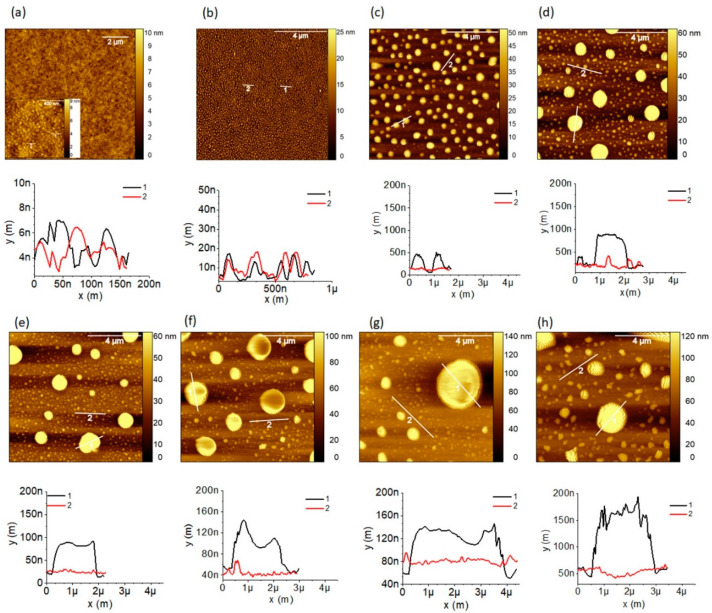
Atomic force microscopy (AFM) images (tapping mode) and the corresponding lateral profile of Cs_2_CO_3_ films printed using the ink with 2 mg/mL concentration at different resolutions: (**a**) 100 dpi, (**b**) 200 dpi, (**c**) 300 dpi, (**d**) 400 dpi, (**e**) 500 dpi, (**f**) 600 dpi, (**g**) 700 dpi, and (**h**) 800 dpi. The scanned area is 10 μm × 10 μm. The inset AFM image in (**a**) is scanned over 1 μm × 1 μm. x(m) represents profile line width and y(m) represents profile height.

To characterize the morphology of printed Cs_2_CO_3_ layers we used the AFM technique. The AFM raw images obtained were processed and analyzed using open source software, Gwyddion. The AFM images of the printed films were scanned over the 10 µm × 10 µm area, and in case films printed at 100 dpi, a small area of 1 µm × 1 µm was also inspected. Figure 6 presents AFM images and the lateral profile of Cs_2_CO_3_ layers printed at various resolutions. Lateral profile was taken at two locations for each image. The Cs_2_CO_3_ film obtained at 100 dpi appear amorphous when scanned over the area of 10 μm × 10 μm; however, when it is probed over a small area (1 μm × 1 μm, inset of Figure 6a), we observed closely packed nanoparticles/crystals whose size is approximately 50 nm (see line profile, Figure 6a). The films obtained at 200 dpi clearly show almost spherical particles/crystals, densely packed and of similar size of approximately 200 nm (Figure 6b). The films printed at 300 dpi and higher resolution comprised of randomly distributed nano/micro-sized particles, as seen in Figure 6c–h, which we believe is because of crystallization. Because the Cs_2_CO_3_ function as electron injection layer only if it is thin, therefore, we assume that the low thickness regions are ensuring the injection of the electrons.

### 3.6. Performance of PLEDs with Different Cathode Systems

**Figure 7:**
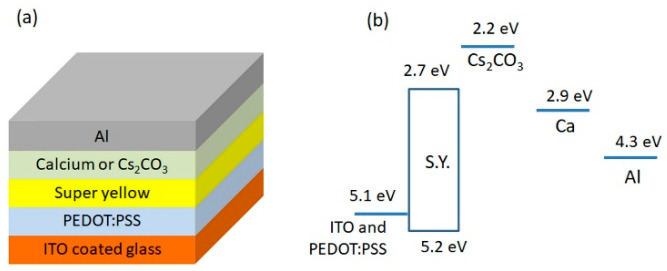
Schematic representation of (**a**) PLED structure and (**b**) energy levels of functional layers [59].

We have fabricated PLEDs with printed Cs_2_CO_3_ covered by evaporated Al as a cathode. Figure 7a illustrates schematically the device structure and energy levels of PLEDs components. Furthermore, to compare the performance of printed Cs_2_CO_3_, we have fabricated PLEDs with different cathode systems: only Al; evaporated Ca/Al; evaporated Cs_2_CO_3_/Al, and spin-coated Cs_2_CO_3_/Al (Al was always vacuum evaporated).

**Figure 8:**
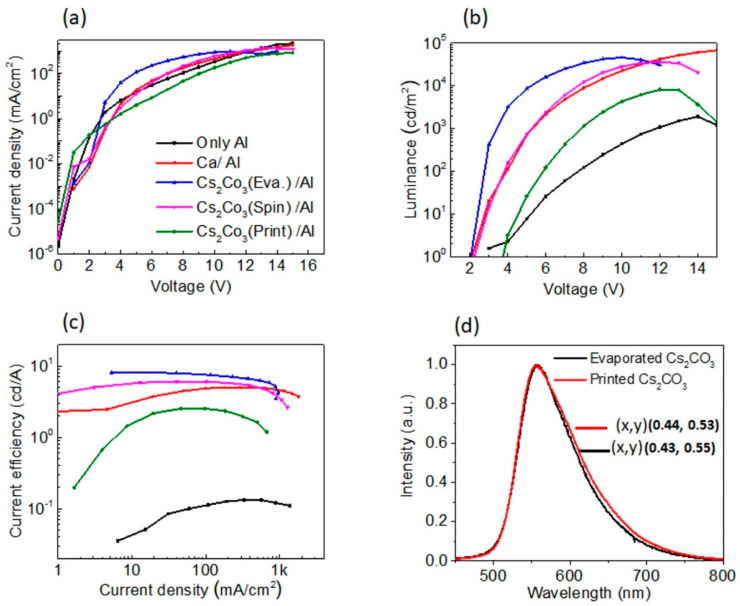
Characteristics of PLEDs with different cathode systems: (**a**) current density vs. voltage; (**b**) luminance vs. voltage; (**c**) current efficiency vs. current density; and (**d**) electroluminescence spectra.

PLEDs characteristics: current density vs. voltage, luminance vs. voltage, and current efficiency vs. current density are presented in Figure 8. Table 3 lists the basic PLEDs performance parameters: turn-on voltage, maximum luminance, and maximum current efficiency. The PLEDs fabricated only with Al as cathode showed poor performance with a maximum luminance of 1900 cd/m^2^, the maximum current efficiency of 0.13 cd/A, and a turn-on voltage of 4 V because of the high energy barrier (1.6 eV) for electrons, as depicted in Figure 7b. Such a high injection barrier causes a low electron injection leading to the imbalance of electrons and holes density inside the emissive layer, resulting in weak emission and high turn-on voltage. When the calcium was added between Al and SY layers, the device performance improved drastically. The enhancement is undoubtedly due to low work function metal (Ca), which improves electrons’ injection into the EML. The maximum luminance, maximum current efficiency, and turn-on voltage of these PLEDs were 51,000 cd/m^2^, 5.0 cd/A, and 2.5 V, respectively. When the evaporated Cs_2_CO_3_ was inserted between Al and SY, further increase in the current efficiency was observed, which indicates Cs_2_CO_3_ interlayers is more efficient than Ca. It has been reported that a thin layer cesium carbonate on semiconducting layer creates a n-doping effect resulting in the improved electron injection property [35]. The spin-coated Cs_2_CO_3_ also forms efficient electron injection interlayer and PLEDs displayed slightly higher current efficiency than the PLEDs with Ca/Al, indicating the potential application for printable electronic devices. Our results are similar to Super Yellow-based PLEDs previously recorded in literature [60,61].

The PLEDs with printed Cs_2_CO_3_ performed better than the PLEDs without Cs_2_CO_3_ showing a fourfold increase higher brightness and twenty-fold increase in current efficiency. However, their performance was lower than PLEDs composed of evaporated Cs_2_CO_3_ or spin-coated Cs_2_CO_3_ (see Table 3). The reason could be the layer morphology because the spin coated or the evaporated Cs_2_CO_3_ films are smooth, without crystalline particles (Figure S4b,c). Furthermore, oxygen plasma treatment of the SY layer (done to improve the Cs_2_CO_3_ ink wetting) might degrade the SY layer. To check this hypothesis, we fabricated the PLEDs with oxygen plasma-treated SY film and untreated SY film, and for both the devices, Ca/Al was used as the same cathode system. The device performances are shown in Table 4 (the characteristics plots are shown in Figure S5). It can be seen that there is an insignificant decrease in the parameters of the plasma-treated device compared to the device without oxygen plasma treatment. In conclusion, we assume that formation of micro/nanoparticles can be considered as the main factor for declining printed Cs_2_CO_3_ device performance.

Figure 8d shows the normalized EL spectra of PLED with printed Cs_2_CO_3_ and of the reference PLEDs with the evaporated Cs_2_CO_3_. The emission peak of PLED with printed Cs_2_CO_3_ is at λ = 546 nm, which corresponds to yellow color, and it matches the emission spectrum of the PLEDs with evaporated Cs_2_CO_3_. The CIE coordinate (x,y) (taken at c.a. 6000 cd/m^2^) of printed and evaporated PLEDs were (0.44, 0.53) and (0.43, 0.55), respectively, which indicates that there is only a little variation in the emitted color. Thus, the solvent used for the Cs_2_CO_3_ ink formulation did not affect SY’s luminescence properties.

### 3.7. Application: Patterning of Emission Area (EA) of PLEDs

**Figure 9:**
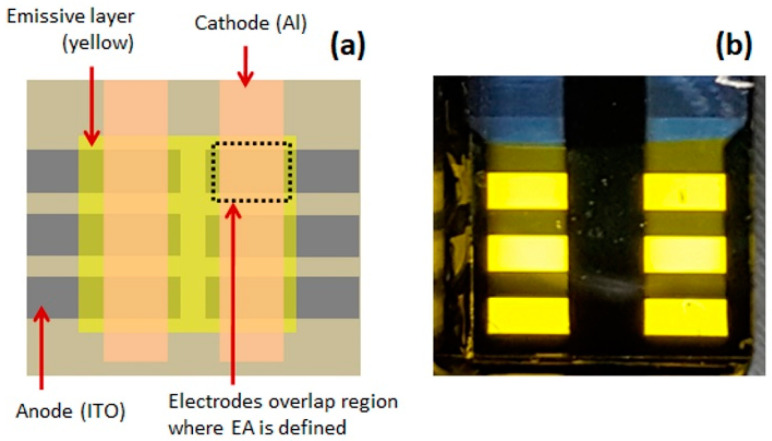
(**a**) Schematic shows an emission area patterning by the overlapping electrode and (**b**) light emission from PLEDs fabricated with an electrode patterning process. The emission area of OLEDs is 3 mm × 1.5 mm.

Usually, the PLEDs’ emission areas are defined by overlapping the bottom and top electrodes, as shown in Figure 9a. In such a method, generally, the bottom ITO electrode is patterned by lithography, and the top Al cathode is patterned using a metal mask. The PLEDs fabricated in this way are shown in Figure 9b. This technique requires careful ITO etching or/and unique patterned metal masks for top electrode deposition to obtain different EA. Alternatively, as discussed in the Introduction, the EA can be defined by screen printing or spray coating a dielectric layer (to form the light-blocking layer) over the emissive layer. However, such a process also requires masks, which limit the resolution of the EA. Moreover, it adds an extra step, which increases PLEDs’ production complexity.

**Figure 10:**
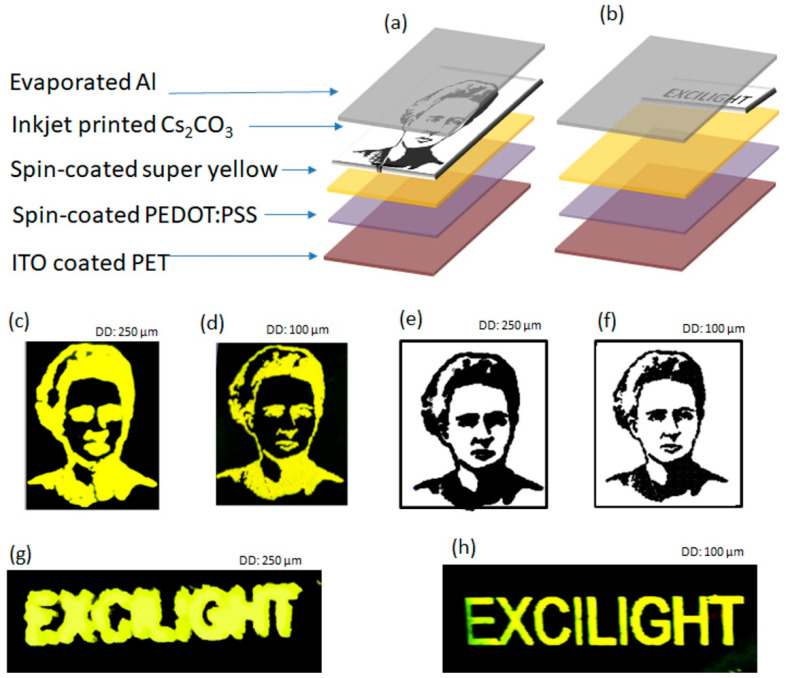
Schematic structure of PLEDs where an emission area is defined by printed Cs_2_CO_3_ pattern: (**a**) Marie Skłodowska Curie portrait and (**b**) text “EXCILIGHT”. Pictures of PLEDs emitting light under applied voltage of 6V printed with (**c**) 250 µm drop diameter and (**d**) 100 µm drop diameter. Simulated image (**e**) 250 µm drop diameter and (**f**) 100 µm drop diameter. The text “EXCILIGHT” printed with (**g**) 250 µm drop diameter and (**h**) 100 µm drop diameter. The emission area of OLEDs of Marie Skłodowska Curie portrait was 15 mm × 10 mm and of the “EXCILIGHT” word is 15 mm × 3 mm.

Here, we demonstrated an effective and simple method for EA patterning. The main idea of this process is that the emission area is controlled by the EIL. This means that the PLEDs emit light only from the regions where the EIL is deposited, while the areas without EIL remain dark. Hence, this approach is free from patterning electrodes or printing dielectric layers and, therefore, has fewer fabrication steps; besides, the inkjet technique enables high-resolution image printing. To demonstrate this idea, we have fabricated PLEDs on ITO-coated glass substrate and PET substrate. Figure 10a,b show the device structure. The fabrication process involves the spin coating of the PEDOT:PSS and the SY films subsequently on an unpatented ITO-coated substrate. Next, the portrait of “Marie Curie” and the text “EXCILIGHT” were printed using Cs_2_CO_3_ ink on separate samples and, followed by the deposition of Al over the entire area (without mask) using the thermal evaporation process. It can be clearly seen from the Figure 10c,d,g,h that PLEDs light emits only from the regions where the Cs_2_CO_3_ is deposited. These results confirm the possibility of EA patterning via wisely printing the EIL.

For any practical application, in addition to the easy fabrication process, the details of the printed images are very important. Here we have shown that the drop diameter plays an important role in production by printing a high-quality Marie Skłodowska Curie portrait. The impact of the drop diameter on the quality of the Marie Skłodowska Curie portrait is shown in the Figure S6, which is simulated through the PiXDRO simulator. The image obtained at a drop diameter of 100 µm fairly matches with a quality of input bitmap image. The image obtained below 100 µm drop diameters suffers from inadequate coverage, whereas at higher drop diameter, the image details deteriorated.

To validate the simulation results, we printed the portrait of Marie Skłodowska Curie as EIL at two different drop diameters: 250 µm and 100 µm and compared them with the simulated images. The drop diameter was managed by controlling the droplet volume regulated by the waveform that drives the printhead. The waveform images that generate a drop volume of 10 pL (leads to 100 µm drop diameter) and 27 pL (leads to 250 µm drop diameter) are shown in the Supplementary Figure S7. Figure 10c,e show the printed images and the simulated image obtained at a drop diameter of 100 µm, respectively. Figure 10d,f show the printed images and the simulated image obtained received at a drop diameter of 250 µm, respectively. One can see that the printed PLEDs’ details match the simulated images. Similarly, PLEDs with the text “EXCILIGHT” printed at 100 µm exhibits more details than images obtained with 250 µm; see Figure 10g,h. Thus, our results indicate that it is possible to define the quality of the patterned emission area through controlling the drop diameter, which is a very simple process.

## 4. Conclusions

We have demonstrated the possibility of inkjet printing of cesium carbonate layers, which can be used as the electron injection interlayer for PLEDs applications. The cesium carbonate ink was formulated by dissolving the Cs_2_CO_3_ in 2-etheoxyethanol, a straightforward ink formulation process. We have optimized the waveform parameters that drive the piezo-head for this ink and obtained a stable spherical drop necessary for printing good-quality films. While optimizing the printing parameters, we have examined the influence of printing resolution on the surface morphology of the Cs_2_CO_3_ film. The results show that printed films are composed of micro/nano-sized particles/crystals distributed randomly. It is noteworthy to mention that the PLEDs with printed Cs_2_CO_3_ layers offer 20 times higher maximum current efficiency (2.5 cd/A) and four times higher maximum luminance (8000 cd/m^2^) than the PLEDs without Cs_2_CO_3_ layer (0.12 cd/A and 1900 cd/m^2^). These results indicate that the inkjet-printed Cs_2_CO_3_ layers work efficiently as an efficient electron injection interlayer in PLEDs.

Furthermore, we demonstrated a novel approach for patterning of the emission area of PLEDs. The procedural steps of this technique are much simpler to execute compared to other methods. Our methods can be employed to produce PLEDs with various complex shapes for printed electronic applications such as smart packaging, advertising, signage, etc. Moreover, it is worth mentioning that the Cs_2_CO_3_ is a low-cost material.

This study is the first example, to the best of our knowledge, where the inkjet-printed Cs_2_CO_3_ layers were used as electron injection interlayers for PLEDs applications and for patterning the emission area of PLEDs. The remaining problem for the future studies is the better control the morphology of the printed Cs_2_CO_3_.

## Figures and Tables

**Table 1:** Physical properties of the Cs_2_CO_3_ ink.

Cs_2_CO_3_ Ink Composition	Viscosity (cP) at 25 °C	Surface Tension (dyne/cm)	Density (mg/mL)	Z Number
2 mg/mL in 2-Ethoxyethanol	1.3	18	932	22.4

**Table 2:** Maximum luminance and maximum current efficiency of polymer organic light-emitting diodes (PLEDs), where EIL was printed with various Cs_2_CO_3_ ink concentrations with resolution 500 dpi.

Concentration of Cs_2_CO_3_ (mg/mL) in ink	Maximum Luminance (cd/m^2^)	Maximum Current Efficiency (cd/A)
No EIL	1800 ± 100	0.11 ± 0.02
0.5	5600 ± 500	0.5 ± 0.1
1	7300 ± 500	0.75 ± 0.15
2	7500 ± 600	2.2 ± 0.3
3	3200 ± 500	1.2 ± 0.2

**Table 3:** Turn-on voltage, maximum luminance and maximum current efficiency of PLEDs based on SY with different cathode systems.

Cathode System	Turn-on Voltage (V) @ 1 cd/m^2^	Maximum Luminance (cd/m^2^)	Maximum Current Efficiency (cd/A)
Only AL	4.0–5.0	1800 ± 100	0.11 ± 0.02
Evaporated Ca/Al	2.5	48,000 ± 3000	4.8 ± 0.2
Evaporated Cs_2_CO_3_/Al	2.5	43,500 ± 1500	7.9 ± 0.2
Spin- coated Cs_2_CO_3_/evaporated Al	2.5	34,500 ± 1200	5.7 ± 0.3
Printed Cs_2_CO_3_/ evaporated Al	4.0–5.0	7500 ± 600	2.2 ± 0.3

**Table 4:** Turn-on voltage, maximum luminance and maximum current efficiency of two PLEDs, one with plasma-treated SY layer and second with non-treated SY layer.

PLEDs Structure	Turn-on Voltage (V) @ 1 cd/m^2^	Maximum Luminance (cd/m^2^)	Maximum Current Efficiency (cd/A)
ITO/PEDOT:PSS/plasma treated SY/Ca/Al	2.5	44,000 ± 1200	4.2 ± 0.2
ITO/PEDOT:PSS/pristine SY/Ca/Al	2.5	48,000 ± 3000	4.8 ± 0.2

## Data Availability

not applicable.

## Supplementary Materials

The following are available online at https://www.mdpi.com/2073-4360/13/1/80/s1, Figure S1: Pictures captured from the fiducial camera, which is integrated to the inkjet printed: (a) wet and (b) dried Cs2CO3 films printed on non-treated Super Yellow surface, (c) wet and (d) dried Cs2CO3 films printed on oxygen plasma treated Super Yellow surface. Figure S2. Characteristics of PLEDs fabricated with different concentrations of Cs_2_CO_3_ in the ink and with resolution 500 dpi: (a) current density vs. voltage; (b) luminance vs. voltage; and (c) current efficiency vs. luminance. Figure S3: Characteristics of PLEDs fabricated with the Cs_2_CO_3_ ink with 2 mg/mL concentration using different print resolutions (a) current density vs. voltage characteristics (b) luminance vs. voltage and (c) current efficiency vs. luminance characteristics. Figure S4: AFM images (tapping mode) and line profile of (a) pristine SY film (b) evaporated Cs_2_CO_3_ on SY film (c) spin coated Cs_2_CO_3_ on SY film. x(μm) represents profile line width and y (nm) represents profile height. Figure S5: Characteristics of PLEDs fabricated with plasma treated SY and non-plasma treated SY: (a) current density vs. voltage; (b) luminance vs. voltage; and (c) current efficiency vs. current density. Figure S6: Image of input picture and the images obtained after the simulation at different drop diameter (DD): 10 µm, 50 µm, 100 µm, 200 µm, 250 µm. The resolution of the images was 250 dpi and the Cs_2_CO_3_ ink concentration was 2 mg/mL. Figure S7: Waveform that which generates drop volume of (a) 10 pL and (b) 27 pL for the Cs_2_CO_3_ ink concentration 2 mg/mL.
