# Supplementary material for: Inkjet Printing of an Electron Injection Layer: New Role of Cesium Carbonate Interlayer in Polymer OLEDs
Source: Polymers (Basel). 2020 Dec 28;13(1):80. doi: 10.3390/polym13010080 (PMC7795449; doi:10.3390/polym13010080)
Supplement: Supplementary file 1 [file polymers-13-00080-s001.pdf]

Inkjet Printing of an Electron Injection Layer: New role of cesium carbonate interlayer in Polymer LEDs

Amruth C, Beata Luszczynska*, Wassima Rekab, Marek Zdzislaw Szymanski and Jacek Ulanski*

Department of Molecular Physics, Faculty of Chemistry, Lodz University of Technology, 90-924, Lodz, Poland; amruth.c@ucalgary.ca (A.C); beata.luszczynska@p.lodz.pl (B.L.); wassima.rekab@p.lodz.pl (W.R.); marek@marekszymanski.com (M.Z.S.); jacek.ulanski@p.lodz.pl (J.U.)

*Correspondence: jacek.ulanski@p.lodz.pl; beata.luszczynska@p.lodz.pl ; tel.: +48-42 631 32 16

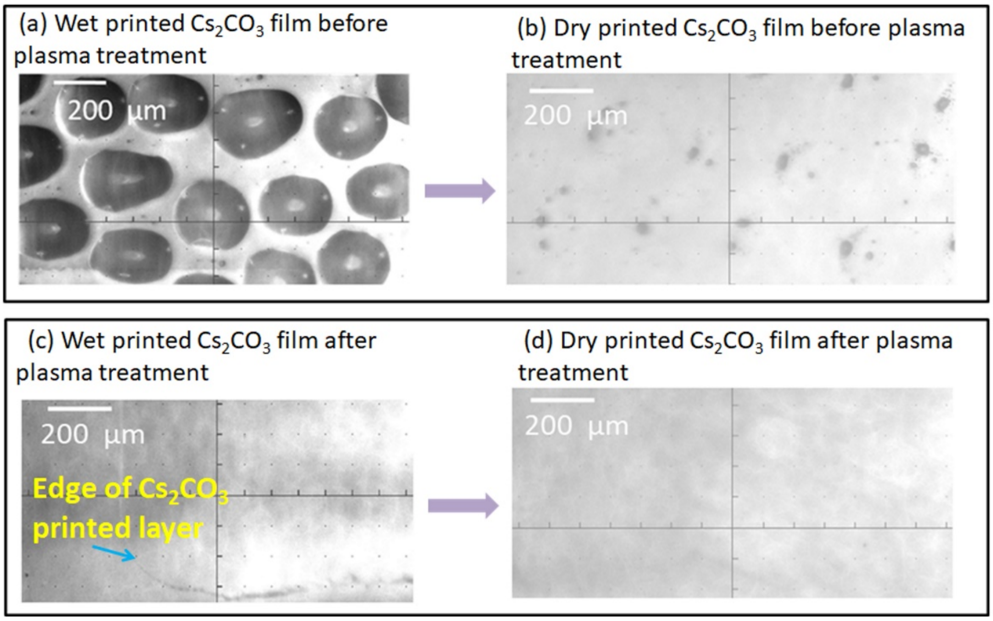

Figure S1. Pictures captured from the fiducial camera, which is integrated with the inkjet printer: (a) wet and (b) dried Cs_2CO_3 films printed on non-treated Super Yellow surface, (c) wet and (d) dried Cs_2CO_3 films printed on oxygen plasma treated Super Yellow surface.

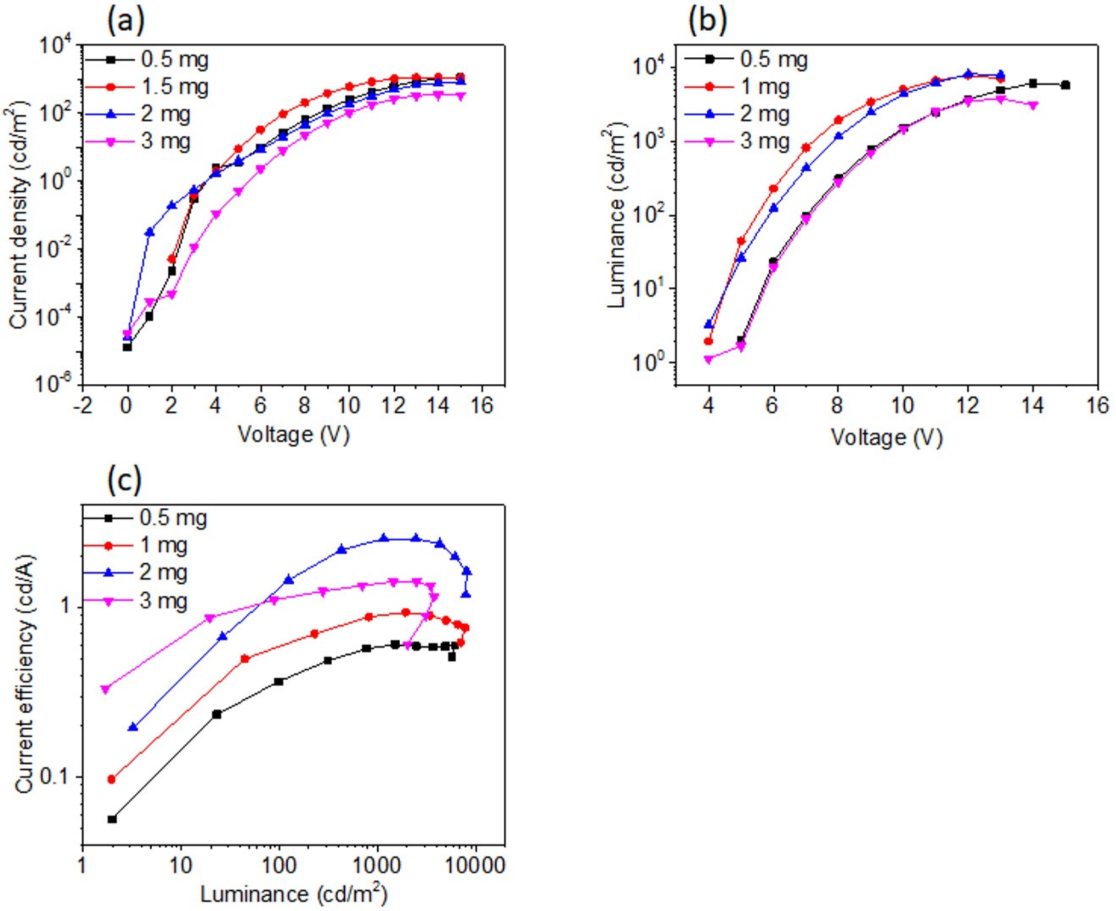

Figure S2. Characteristics of PLEDs fabricated with different concentrations of Cs_2CO_3 in the ink and printed with resolution 500 dpi: (a) current density vs. voltage; (b) luminance vs. voltage; and (c) current efficiency vs. luminance.

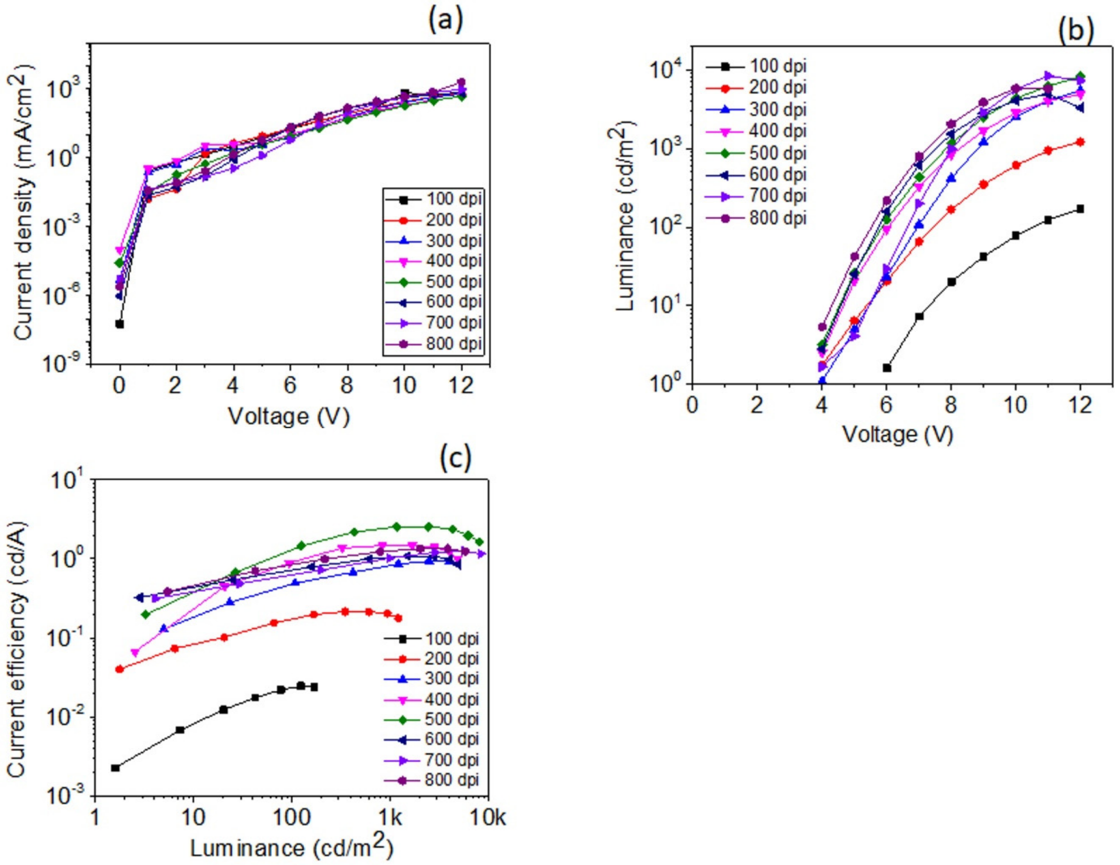

Figure S3. Characteristics of PLEDs fabricated with the Cs_2CO_3 ink with 2 mg/mL concentration using different print resolutions (a) current density vs. voltage characteristics (b) luminance vs. voltage and (c) current efficiency vs. luminance.

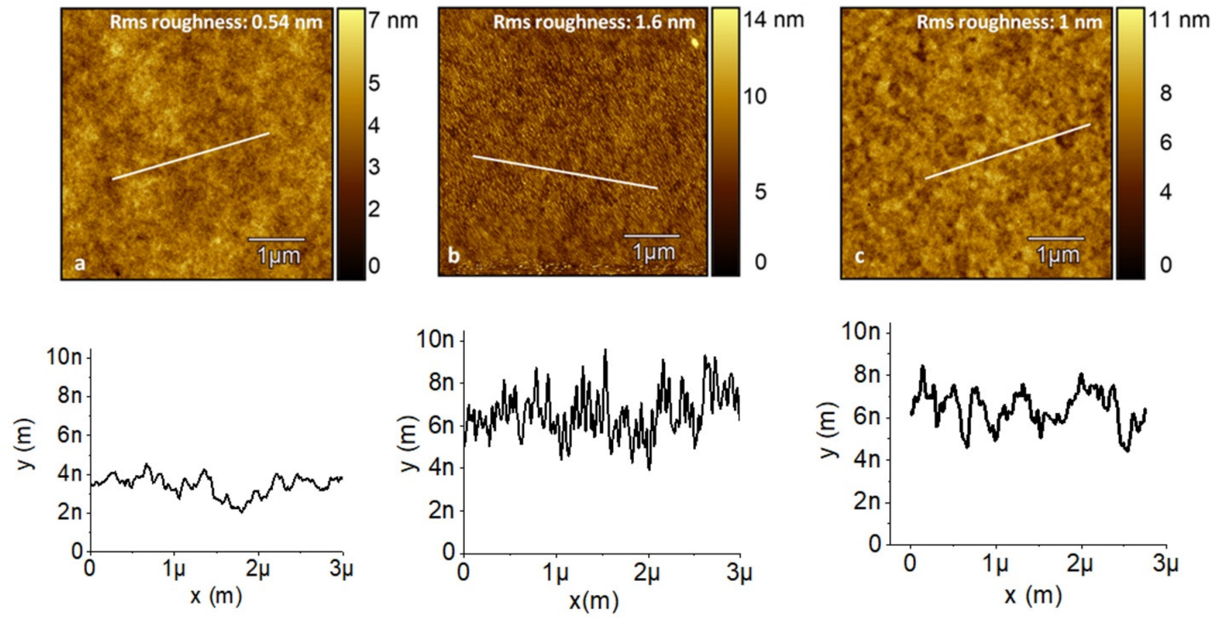

Figure S4. AFM images (tapping mode) and line profile of (a) pristine SY film (b) evaporated Cs_2CO_3 on SY film (c) spin coated Cs_2CO_3 on SY film. x (m) represents profile line width and y (m) represents profile height.

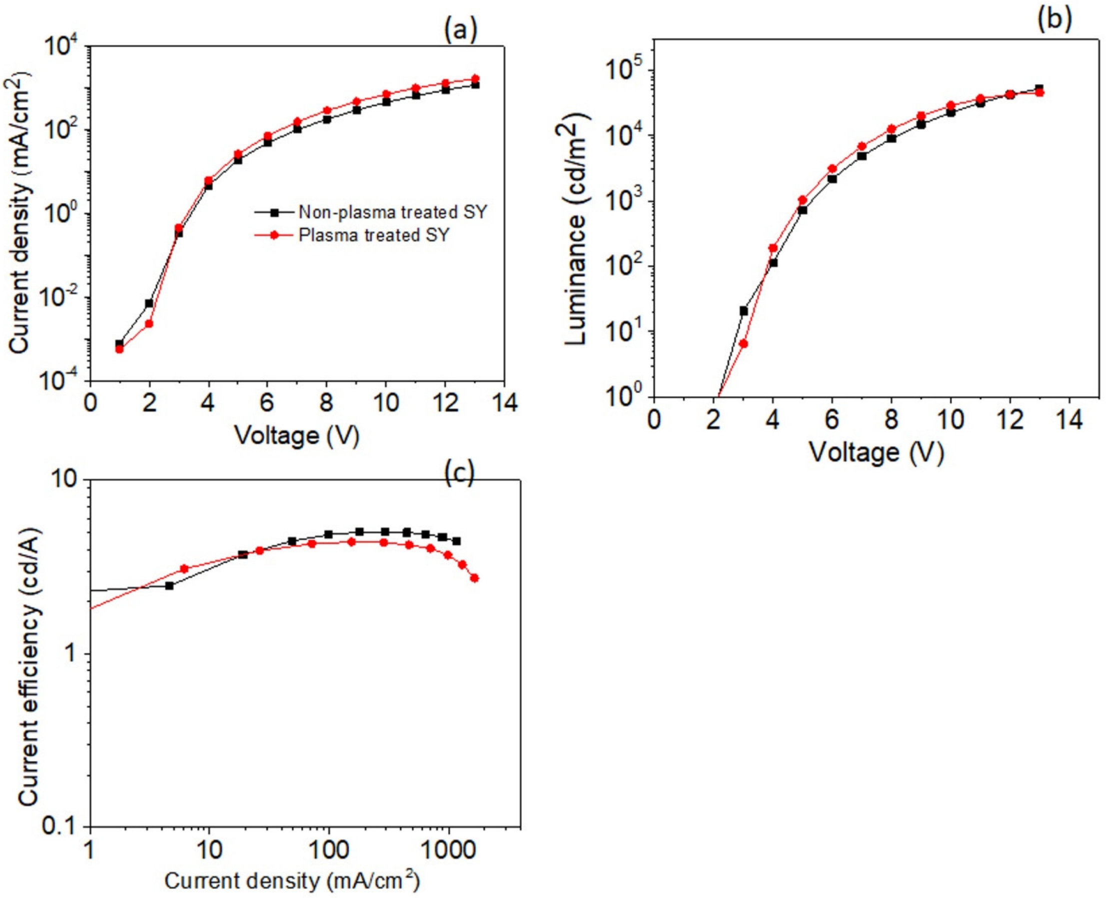

Figure S5. Characteristics of PLEDs fabricated with plasma treated SY and non-plasma treated SY: (a) current density vs. voltage; (b) luminance vs. voltage; and (c) current efficiency vs. current density.

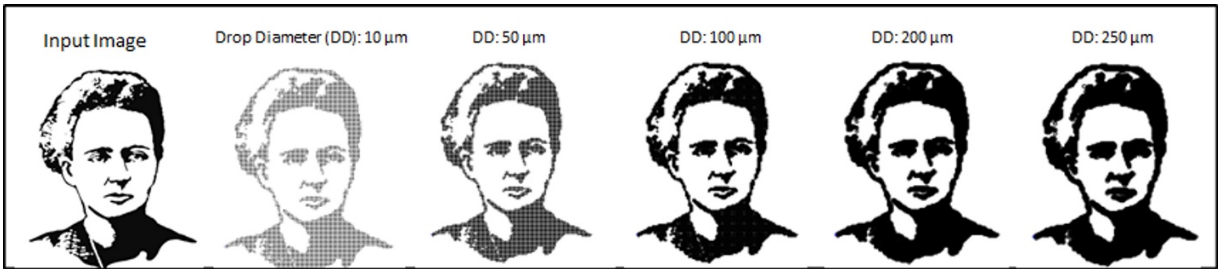

Figure S6. Image of input picture and the images obtained after the simulation at different drop diameter (DD): 10 µm, 50 µm, 100 µm, 200 µm, 250 µm. The resolution of the images was 250 dpi and the Cs_2CO_3 ink concentration was 2 mg/mL.

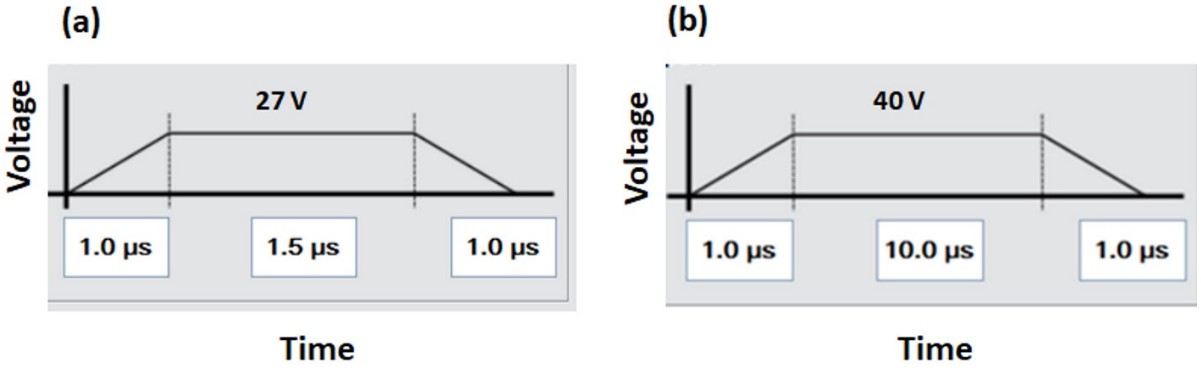

Figure S7. Waveform that which generates drop volume of (a) 10 pL and (b) 27 pL for the Cs_2CO_3 ink concentration 2 mg/mL.
